# Detraining Effects Prevention: A New Rising Challenge for Athletes

**DOI:** 10.3389/fphys.2020.588784

**Published:** 2020-10-15

**Authors:** Michele Girardi, Andrea Casolo, Stefano Nuccio, Chiara Gattoni, Carlo Capelli

**Affiliations:** ^1^Department of Psychology, Center for Brain Science, University of Essex, Colchester, United Kingdom; ^2^Department of Bioengineering, Imperial College London, London, United Kingdom; ^3^Department of Movement, Human and Health Sciences, University of Rome “Foro Italico”, Rome, Italy; ^4^Endurance Research Group, School Sport and Exercise Sciences, University of Kent, Chatham, United Kingdom; ^5^Department of Neuroscience, Biomedicine and Movement Sciences, University of Verona, Verona, Italy

**Keywords:** COVID-19, injury prevention, endurance performance, neuromuscular function, training strategies, detraining

## Introduction

The newly discovered coronavirus (SARS-CoV-2) has caused an infectious disease of pandemic proportion called coronavirus 2019 disease (COVID-19). The absence of an effective vaccine for the COVID-19 disease has led many National and International authorities to take some prompt strict measurements to reduce the risk of infection, including closing non-essential activities and forcing individuals to stay at home. Accordingly, several sport events have been canceled and/or postponed and, hundreds of thousands of amateur and professional athletes worldwide have abruptly been forced to train at home. As a consequence, athletes had to face an unprecedented and relatively long-term reduction or cessation in their training routine along with a substantial cutting of their physical daily activities. Such changes may result in a significant decay of the quantity and worsening of the quality of training stimuli, making athletes exposed to some potential levels of detraining (i.e., “*partial or complete loss of training-induced anatomical, physiological and performance adaptations”*; Mujika and Padilla, 2000b) and to increased risks of injury. Thus, sport scientists, coaches and exercise physiologists worldwide had to deal with a novel challenge consisting in how to minimize potential detraining effects induced by home confinement.

Detraining prevention can be defined as a set of physical training strategies aimed at limiting and/or counteracting detraining effects. The prevention of detraining processes is a fairly new concept, which so far has mainly been addressed in the field of occupational physiology. For instance, a large body of literature has focused on understanding strategies used to counteract detraining processes associated with prolonged exposure to microgravity in astronauts (Hargens et al., 2013; Hackney et al., 2015). Some studies have also investigated the effects of reduced training stimuli on physical performance in athletes (Neufer, 1989; Rietjens et al., 2001; García-Pallarés et al., 2009, 2010; Ormsbee and Arciero, 2012; Joo, 2018). However, these are limited and controversial and they can only provide indirect information on detraining prevention strategies. For example, whereas 21 days of training-stimuli reduction (continuous and intermittent endurance training, 3 days/week) seem to counteract detraining effects (Rietjens et al., 2001), impairments on endurance performance, resting metabolic rate, body weight and composition have been found following 35–42 days of light-moderate exercise (<6.0 METS, 3 days/week) (Ormsbee and Arciero, 2012). Moreover, the training strategies used in these studies are often non-compatible with home-based-training settings as athletes might not have easy access to specific tools/equipment and sport facilities. Yet, the most effective training frequency, volume and intensity as well as exercise modalities to use for preventing detraining are still unknown. Therefore, considering the lack of a COVID-19 vaccine and the possibility that similar home-confinement scenarios would present again, identifying the most effective strategies to minimize detraining effects represents a current priority. To help with this purpose, this brief report illustrates the potential morphological, physiological and functional changes induced by home-confinement. Additionally, specific issues associated with injured athletes have also been discussed.

## Potential Morphological, Physiological and Functional Changes Due to COVID-19-Induced Detraining

To identify an optimal detraining prevention strategy, it is important to determine the main detraining-induced morphological, physiological, and functional adaptations. Training cessation leads to changes in VO_2max_ during both short- (≤4 weeks) and long-term (≥4 weeks) periods (Mujika and Padilla, 2000a,b). Reductions in VO_2max_ seem to be progressive and proportional to individuals' fitness level (Mujika and Padilla, 2000a,b). However, although the VO_2max_ magnitude is often considered an indirect marker of endurance capacity, its changes may not directly be correlated to endurance performance alterations. For example, it has been found that expansions in blood volume can partially reestablish VO_2max_ losses following a period of training cessation; nonetheless, this manipulation was not able to compensate for endurance performance decrements (Coyle et al., 1986). Moreover, 4 weeks of training cessation have been shown to decrease performance during a time to exhaustion test (TTE) without affecting VO_2max_ in well-trained endurance athletes (Madsen et al., 1993; Pedlar et al., 2018). Impairments in endurance performance have also been found during 12–35 days of training cessation in both running and cycling incremental tests (Coyle et al., 1986; Houmard et al., 1992, 1993), a Yo-Yo intermittent-test (Joo, 2018), a 3,000-m running time trial (Pereira et al., 2016) and a cycling TTE (Madsen et al., 1993).

At the muscle level, the relatively short half-life of mitochondrial proteins (~1 week) (Hood, 2001) may cause decrements in mitochondrial function and capacity after a short period of training cessation. In line with this, decrements in muscle oxidative capacity (Coyle et al., 1985; Gjøvaag and Dahl, 2008) and reductions in mitochondrial enzyme activities (Coyle et al., 1985; Wibom et al., 1992) have been found after few days/weeks of training cessation. Non-systematic changes have been observed in glycolytic enzymes quantity and activity (Mujika and Padilla, 2001) whereas, reductions in muscle capillary density have been reported after 4–8 weeks of training cessation (Klausen et al., 1981).

Training stimuli cessation and the consequent decline in plasma volume, which may occur after 2 days of inactivity (Thompson et al., 1984; Cullinane et al., 1986), lead to a reduced cardiac preload, which in turn triggers a series of rapid morphological and functional cardiac remodeling (Martin et al., 1986; Spence et al., 2011; Pedlar et al., 2018). In line with this, impairments in maximal cardiac output (Q_max_) have been found after 12 days of inactivity due to a 10% decrement in exercise stroke volume and 4% increment in maximal heart rate (Coyle et al., 1984). Similar results have also been observed following a period of training cessation and head-down tilt bed rest during both maximal (Coyle et al., 1984; Pedlar et al., 2018) and submaximal exercise (Coyle et al., 1986; Capelli et al., 2008). Such reductions in Q_max_ are critical as they may highly contribute in declining the maximal oxygen delivery capacity.

Training cessation can also markedly affect the volitional force-generating capacity of human skeletal muscles, which is the result of an interplay of neural and morphological factors including muscle cross-sectional area, muscle architecture, muscle fiber type, tendon properties and neural drive to the spinal-motor pool (Bosquet et al., 2013). It has been reported that all these physiological factors involved in volitional force-generation mechanisms can be affected by 8–12 weeks of training cessation, with maximal muscle force decrements predominantly caused by neural alterations in the initial weeks of training cessation and by morphological ones when the period of inactivity exceeds several weeks (Bosquet et al., 2013). For instance, a significant decline in maximal isometric force (~7.5%) in subjects accustomed to strength training has been found after 8 weeks of training stoppage. Interestingly, this force decrement was coupled with decreases (~5–12%) in maximal electromyographic activity reflecting a precocious reduction of muscle activation (Häkkinen and Komi, 1983). In another study from the same group, marked declines in strength performance (~12%) were accompanied by a reduction in the FT/ST muscle fiber area ratio (from 1.11 to 1.04), likely as a result of a tendency toward higher oxidative muscle fiber populations, as well as by a reduction of muscle mass after 8 weeks of training cessation, i.e., muscle atrophy (Häkkinen et al., 1981). Longer periods of detraining (12 weeks) were also accompanied by substantial decreases of the mean muscle fiber areas of both fiber types (Häkkinen et al., 1985). In line with these studies, muscle atrophy and other detraining-induced morphological changes in muscle fiber distribution and architecture (Coyle, 1988) and/or FT cross-sectional area (Bangsbo and Mizuno, 1988; Allen, 1989; Amigó et al., 1998) have been consistently reported in more recent investigations for athletes of different disciplines such as endurance runners, cyclists, soccer and rugby players, following 3–8 weeks of training cessation. Conversely, despite novel evidences have arisen from bed rest studies showing that chronic inactivity induces muscle denervation and damage to the neuromuscular junction (Narici et al., 2020), the understanding of training-cessation-induced neural changes, particularly at the single motor unit level is still limited. Moreover, prolonged exposure to mechanical unloading may also cause impairments in tendon structures and properties (Frizziero et al., 2016) as well as at the soft-tissue level (e.g., articular capsule, cartilage, ligaments, synovium). Specifically, compromised tendon reactions to a load application (Frizziero et al., 2016; Paoli and Musumeci, 2020) and a dramatic decrement in cartilage lubrication and nutrition (Castrogiovanni et al., 2019) in response to inactivity have been recently documented. Taken together, the rate at which these morphological and physiological remodeling adaptations occur underlines the importance of movement and exercise to preserve not only the integrity of the muscles, but also of the neural circuits upstream, tendons and joint structures in situations of reduced-training stimuli and mechanical unloading, such as the COVID-19 home confinement.

Regaining a pre-detraining status is also essential for athletes. As effective training programs do, reconditioning training programs also need to match training principles (Garber et al., 2011). The time required to recover pre-detraining neuromuscular and cardiorespiratory levels may highly vary among athletes on the base of several factors, including time of training stimuli cessation or reduction, amount of individual detraining-induced effects, individual fitness levels and sport-specific requirements. For instance, following 20 days of bed rest, VO_2max_, Q_max_, blood plasma volume and heart volume values were recovered after a reconditioning training program ranging from few days to 55 days, where longer periods seem to be required for trained compared to untrained individuals (Saltin et al., 1968). While cardiovascular values may recover in few days (Saltin et al., 1968), longer training periods might be required to regain pre-detraining levels of muscle oxidative capacity and function (Skattebo et al., 2020). Due to the heterogeneity effects of detraining and training, it is extremely important to perform a battery of sport-specific tests aimed at evaluating the individual detraining status for planning an effective and safer return to sport activities. Sports-specific tests should also be performed to check the efficacy of the reconditioning training program. Importantly, all the stakeholders (e.g., coaches, athletes and medical staff) need to be involved for planning effective and safer reconditioning training programs before, during and after the process itself.

## Injured Athlete During The COVID-19 Enforced Quarantine

A particular case that undoubtedly needs to be considered is the injured athlete in both early and latest rehabilitation and reconditioning stages. In such specific population, the focus of a detraining prevention program shifts from the pursuit of counteracting detraining effects, to the pursuit of finding the best home-based recovery strategy. Indeed, in addition to the potential morphological and physiological detraining effects due to the COVID-19 home confinement, injured athletes might also struggle against detrimental effects associated with the injury itself and with a potential insufficient and/or inappropriate home-based rehabilitation and reconditioning. Although no clear evidence has been provided on this topic, the scientific community suggests insufficient and/or inappropriate rehabilitation and reconditioning stimuli as the main determinants of injury recurrence (Kyritsis et al., 2016). This is particularly relevant for those athletes who suffered from musculoskeletal injuries and needed to readapt either damaged soft-tissues or muscles to loading through a proper neuromuscular rehabilitation.

Injured athletes at their very early stages of rehabilitation may need special attention. The unpredicted closure of sport therapy clinics worldwide could indeed have prevented athletes from optimally tackle the initial impairments related to a musculoskeletal injury. For instance, athletes with reconstructed anterior cruciate ligament would experience an initial inflammatory process on the knee with related pain and swelling, which in turn would cause a substantial inhibition of the quadriceps muscle and an associated dramatic deficit in muscle strength (Rice and McNair, 2010). With the aim of reducing pain and re-establishing knee joint homeostasis, clinicians and health professionals typically strive to fix these issues immediately after surgery and thereby to ensure a progressive joint loading and muscles strength reconditioning in the following stages (Dingenen and Gokeler, 2017). The impossibility to go through these fundamental steps and the absence of adequate professional support, may delay the recovery process and cause long-term problems (e.g., impaired quadriceps function, neuromechanical alterations and compensation strategies) which, in turn, may prevent athletes from returning to their pre-injury physical conditions, and increase the risk of a second knee injury (Hewett et al., 2013). Similarly, also injured athletes in the latest rehabilitation phases would delay their return to sport. This happens because of the impossibility to provide adequate training stimuli aimed at re-gaining sport-specific fitness levels (Buckthorpe, 2019). For all these reasons, the adoption of proper home-based training protocols is pivotal to avoid a delayed or unsafe return to sport. However, recommending potential detraining prevention strategies for injured athletes is extremely challenging as they may vary according to the type and time of injury, individual responses to injury and different external factors (e.g., home setting and equipment availability).

## Conclusion

The COVID-19 pandemic and the consequent forced home confinement have risen a new challenge in the field of sport and exercise sciences, which consists in how to limit and counteract detraining effects among athletes. Training cessation has been shown to negatively affect physical human performance, but very little is known about the effects of training stimuli reduction. Moreover, exceptional situations such as in the case of the COVID-19 enforced quarantine might lead to inadequate rehabilitation and reconditioning programs in injured athletes, which in turn might be translated in a delayed and/or unsafe return to sport. However, researchers have never considered the need of investigating detraining effects prevention yet. Considering the current lack of a COVID-19 vaccine, the strict rules that several countries worldwide are still adopting to stop this pandemic, and the possibility that similar extreme situations would present again, future research in this field is certainly required.

## Author Contributions

MG, AC, SN, CG, and CC contributed to conception and design of the study. MG wrote the first draft of the manuscript. AC, SN, and CG wrote sections of the manuscript. All authors approved the final version of the manuscript and agreed to be accountable for all aspects of the work in ensuring that questions related to the accuracy or integrity of any part of the work are appropriately investigated and resolved. All persons designated as authors qualify for authorship, and all those who qualify for authorship are listed.

## Conflict of Interest

The authors declare that the research was conducted in the absence of any commercial or financial relationships that could be construed as a potential conflict of interest.
